# DeepPilot: A CNN for Autonomous Drone Racing

**DOI:** 10.3390/s20164524

**Published:** 2020-08-13

**Authors:** Leticia Oyuki Rojas-Perez, Jose Martinez-Carranza

**Affiliations:** 1 Department of Computational Science, Instituto Nacional de Astrofísica, Óptica y Electrónica (INAOE), Puebla 72840, Mexico; oyukirojas@inaoep.mx; 2 Department of Computer Science, University of Bristol, Bristol BS8 1UB, UK

**Keywords:** autonomous drone racing, CNN, deep learning

## Abstract

Autonomous Drone Racing (ADR) was first proposed in IROS 2016. It called for the development of an autonomous drone capable of beating a human in a drone race. After almost five years, several teams have proposed different solutions with a common pipeline: gate detection; drone localization; and stable flight control. Recently, Deep Learning (DL) has been used for gate detection and localization of the drone regarding the gate. However, recent competitions such as the Game of Drones, held at NeurIPS 2019, called for solutions where DL played a more significant role. Motivated by the latter, in this work, we propose a CNN approach called DeepPilot that takes camera images as input and predicts flight commands as output. These flight commands represent: the angular position of the drone’s body frame in the roll and pitch angles, thus producing translation motion in those angles; rotational speed in the yaw angle; and vertical speed referred as altitude *h*. Values for these 4 flight commands, predicted by DeepPilot, are passed to the drone’s inner controller, thus enabling the drone to navigate autonomously through the gates in the racetrack. For this, we assume that the next gate becomes visible immediately after the current gate has been crossed. We present evaluations in simulated racetrack environments where DeepPilot is run several times successfully to prove repeatability. In average, DeepPilot runs at 25 frames per second (fps). We also present a thorough evaluation of what we called a temporal approach, which consists of creating a mosaic image, with consecutive camera frames, that is passed as input to the DeepPilot. We argue that this helps to learn the drone’s motion trend regarding the gate, thus acting as a local memory that leverages the prediction of the flight commands. Our results indicate that this purely DL-based artificial pilot is feasible to be used for the ADR challenge.

## 1. Introduction

In 2016, for the first time, a group of researchers proposed to organize the first Autonomous Drone Racing competition (ADR) in the IEEE International Conference on Intelligent and Robotic systems (IROS). This competition was motivated by the popularity of *drone racing*, practiced by hobbyists and professional competitors in the Drone Racing League (https://thedroneracingleague.com). The challenge consisted of developing a drone capable of racing a track autonomously, this is, without pilot or any other human intervention. As an ultimate goal, it is expected that one day an autonomous racing drone will beat a human in a race.

During the ADR competitions, in IROS 2016 and 2017, teams presented drones capable of performing gate detection, based on conventional image processing, localization, mostly based on visual SLAM, and stable flight control [1]. In the ADR of IROS 2018, the use of Deep Learning (DL) became more prominent as it was shown to be more robust for gate detection against varying lighting conditions. This detection was used then to drive the flight control [2,3].

In this sense, a pipeline to address this problem began to dominate, namely: gate detection and visual localization, either global or relative to the gate; vehicle’s state estimation; planning and control [4]. However, at the end of 2019, a competition called Game of Drones (GoD), organized by Microsoft and Stanford University, and hosted at NeurIPS 2019 [5], called for novel approaches where DL was exploited to do more than gate detection. It should be noted that the GoD competition ran in simulation only, using the AirSim simulator. Despite the impressive results obtained by the teams in the GoD competition, the majority of teams also implemented the dominant pipeline mentioned before, including DL for gate detection.

**Figure 1:**
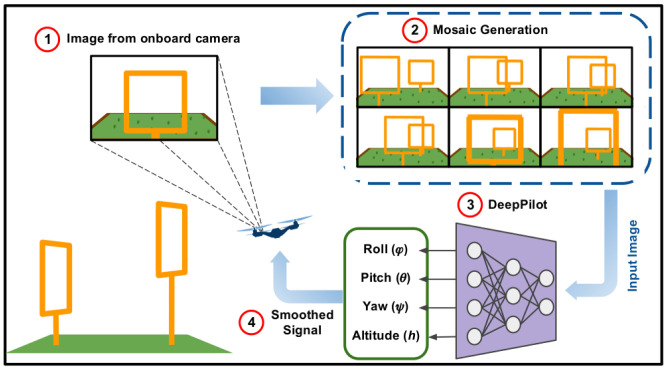
Overview of our approach, it consists of 4 steps: (1) Data acquisition using the drone’s onboard camera; (2) Real-time mosaic generation, consisting of 6 frames; (3) Flight commands prediction using our proposed CNN named DeepPilot, these commands are represented by the tuple (ϕ,θ,ψ,h); (4) Implementation of a filter to smooth the signal. A video illustrating the performance of our proposed DeepPilot can be found at https://youtu.be/Qo48pRCxM40.

Motivate by the above, in this work we present DeepPilot, a monocular approach based on a Convolutional Neural Network (CNN) that receives camera images, from the drone’s onboard single camera; and predicts 4 flight commands, see Figure 1. These commands represent: the angular position of the drone’s body frame in the roll and pitch angles, thus producing translation motion in those angles; rotational speed in the yaw angle; and vertical speed referred as altitude. These flight commands will be referred as the tuple (ϕ,θ,ψ,h) from now onwards. Our approach has been inspired by the work on 6-DoF camera pose (rotation and translation) prediction from a single camera using a CNN architecture called *PoseNet* [6]. We decided to explore a similar approach, aiming at inferring the drone’s flight commands rather than a 6-DoF pose.

As a first attempt, in [7], we re-trained PoseNet to infer only the 2 angular positions for the roll and pitch angles, from a single image. Furthermore, we evaluated the use of a single image against a temporal approach, where 6 consecutive frames are stitched to build a mosaic image that is passed as a single image to the network. We intentionally avoided using a stack of frames as input to the network, since we wanted to use the same architecture input offered by PoseNet, which receives a single image of 3 channels (RGB) as input. We argue that stitching frames in a single image is valid for our problem given that a set of consecutive frames capture a motion trend from the drone’s camera viewpoint and such trend can be appreciated in the mosaic image. In addition, the use of the mosaic avoids adding extra layers, and therefore additional network parameters, that would grow with the number of input images. Our evaluations were carried out in simulation using the Gazebo simulator in simple racetracks with gates always parallel to the drone’s camera plane. The obtained results indicated that it was possible to use PoseNet to control the drone for at least 2 flight commands. We still used a Proportional–Integral–Derivative (PID) controller to control yaw and altitude velocities.

Therefore, based on the results of our previous work, now we present a comprehensive approach with the following contributions:

1. We present a new CNN architecture to predict flight commands from either a single image or a single mosaic image, in both cases captured only with a monocular camera onboard the drone.
2. We present a thorough evaluation in simulated racetracks where gates have different heights and variations in the yaw angle. Opposite to our previous work, where the number of frames to create the mosaic was arbitrarily fixed to 6, we have evaluated the mosaic for when it is composed of 2, 4, 6 or 8 frames and it has been compared against using a single image as input to the network. We aim at showing that temporality is essential for our approach to be successful, since a single image may not be enough for the CNN to predict the drone flight commands. In contrast, we argue that consecutive frames act as a memory that contains a motion trend that can be learned by the network, thus helping to predict adequate flight commands.
3. We show that a successful prediction of the flight command can be achieved if we decouple yaw and altitude commands from roll and pitch. This implies that our model runs 3 parallel branches to infer roll and pitch together, but yaw and altitude separate. This has been inspired by those works on visual SLAM and visual odometry, where orientation and translation are decoupled in order to obtain better prediction results [8,9]. Our experiments indicate that this is also useful to be considered in our approach.
4. The following items are publicly available at https://github.com/QuetzalCpp/DeepPilot.git: our training and test datasets; our trained DeepPilot model; a set of racetrack worlds in Gazebo; and scripts in python to run our model.

In order to present our approach, this paper has been organized as follows: Section 2 offers a concise review of what has been implemented for autonomous drone racing; Section 3 describes our methodology; Section 4 presents our experimental framework; and finally, Section 5 discusses our conclusions and future work.

## 2. Related Work

The autonomous drone racing involves four main challenges: detection, path planning, localization, control for autonomous navigation in a drone racing track. In the first edition of the autonomous drone racing held in IROS 2016 [1,10,11], the majority of the competitors presented solutions where the flight controller relied primarily on gate detection. The racetrack consisted of 24 gates heavily packed in a small space. Even when the gates had different heights and orientations, which made the track challenging, the compact space in between gates made the competing drones to exhibit slow flight. Only for this edition, the gates had attached to them QR codes for teams to use them to identify a gate and its number position in the track. Most of the gate detection algorithms were based on traditional computer vision techniques such as color segmentation and corner detection [12]. The winner team presented a solution based on a set of onboard sensors [10]. The authors used stereo cameras create a depth map relative to the gate. The depth map was used in an obstacle detection and collision avoidance algorithm. The gate was detected using color segmentation and it was used in a control law to command the drone to navigate towards the gate.

For the 2017 IROS autonomous drone racing [1], teams presented more elaborated solutions than in 2016. Interestingly, top tier teams included in their solutions the use of visual localization either based on visual Simultaneous Localization and Mapping (SLAM) or I localization based on a depth camera and the Iterative Closest Point algorithm. Thus, if the drone could be localized in the racetrack, then a waypoint-based solution could be implemented. The winner team proposed a modified version of ORB-SLAM where the camera pose estimates where recovered with metric, even when they used a monocular camera [13]; to recover the scale of the world, the authors used the altimeter, the camera angle, and the planarity of the ground in the race track to define a ray-plane intersection formulation from which a synthetic depth image was generated and used within the RGB-D version of ORB-SLAM [14]; thus, waypoints where strategically placed for the drone to navigate and cross the gates. The runner-up team used a depth camera on board the drone to generate a local point cloud; the latter was aligned against a 3D CAD model of the race track, thus resolving the drone’s localization; waypoints where located some distance before and after the gate for the drone to navigate towards them and to cross them; it must be said that this team achieved the fastest flight in terms of meters per second [1]. Opposite to the use of SLAM, one of the teams proposed a state machine where each state represented a flight behavior [15,16]. The behaviors were defined for different sections in the racetrack. The behavior controller depended on odometry estimates regarding the gate, which implied gate detection. For the latter, the authors implemented a snake gate detection to find the position of the four corners of the gate and then back-project these image positions to 3D points from which the camera pose could be estimated. The snake detection uses the orange color of the gates with a sub-sampling approach [17].

Recent works have proposed to replace the segmentation-based gate detection algorithms with deep learning such as VGG16 [18], Yolo [19], AlexNet [20], MobileNet [21], MobileNet-SSD [22] and Single Shot Detector (SSD) [23,24]. It has been shown that a DL-based detection improves the accuracy of the gate detection and is more robust to changes in the lighting conditions. It is also useful to differentiate more easily the front gate when gate overlaps occur. For instance, in [2,25], the SSD network is used to detect the position of the gate on the image. The center of the bounding box in the detection is used by the controller to align the drone regarding the gate and once aligned, the controller commands the drone to cross the gate.

In a more elaborated work, Kaufmann et al. [3,26], the authors propose a deep network coupled with a Kalman filter to detect the gate, and also to predict the position of the gate regarding the drone. The estimates are used by a predictive controller to follow a flight path planned upon the estimated gate position. In a similar fashion, in [27,28], the authors have adapted PoseNet [6], a CNN network used for camera relocalization, to calculate the position of the drone regarding to a gate. The output of the network provides a vector with the values of *x*, *y* and *z* in meters, which then are used by a PID controller to command the drone, first to center itself regarding to the center of the gate, and once centered, to cross it. In this case, PoseNet is used closer to its original purpose, i.e., as a pose relocalization method, except that using the gate as scene reference.

From the above, the autonomous drone racing solutions presented, use path planning as a principal task, due to the fact that the racetrack is previously known, and combines the gate detection to correct the trajectory. This strategy generates high computational costs, since four processes execute simultaneously, (1) localization, (2) planning, (3) detection and (4) control [4].

In contrast, in a similar challenge, although for autonomous racing cars, some works have pushed for reducing the pipeline mentioned above. For instance, in [29], the authors use a constant velocity, and a CNN fed directly with images, from an onboard camera, to predict the steering angle to drive a car on the road. Similarly, in [30], the authors used the CNN to recognize the road instead and corrected the yaw angle to avoid driving off the way. Likewise, inspired by what has been done for autonomous car driving, the authors in [31] present an approach based on 2 networks. The first one takes an image, captured from the drone’s onboard camera, as input and generates a trajectory defined by 5 points. The second network uses this trajectory and the current drone’s state, defined by its orientation and velocity, and generates throttle, roll, pitch and yaw as output. Note that this work uses the output of the first network, i.e., the trajectory, as an intermediate representation to learn the flight commands, arguably, the dataset and trajectory labelling could induce a strong bias since many trajectories could be assign to the same image. The experiments in this work were also carried out in a simulation environment.

Therefore, motivated by the works discussed in this section, in this work, we present a new CNN architecture to predict the drone’s command controls required by its inner loop controller. These commands are defined by flight commands defined by roll, pitch, yaw and altitude. We have named this network as DeepPilot. Our approach can be seen as a learning by imitation approach; however, we seek to learn the corresponding flight commands directly from a single mosaic image rather than using intermediate representations. Our ultimate goal is to generate a CNN-based model capable of commanding the drone to fly autonomously in a racetrack for the autonomous drone racing challenge. The following sections will describe our approach based on DeepPilot and its validation.

## 3. Proposed Framework

In this section, we describe our DeepPilot architecture, the data acquisition method, the generation of mosaics as input for the DeepPilot network, as well as the filter used to smooth the output of the network. In sum, our approach consists of 4 steps: (1) Image acquisition from the drone’s onboard camera; (2) Generation of the mosaic image, which is updated every 5 frames; (3) Flight commands prediction using DeepPilot; (4) Implementation of a filter for the DeepPilot output, to smooth out the flight commands.

### 3.1. Quadcopter Model

**Figure 2:**
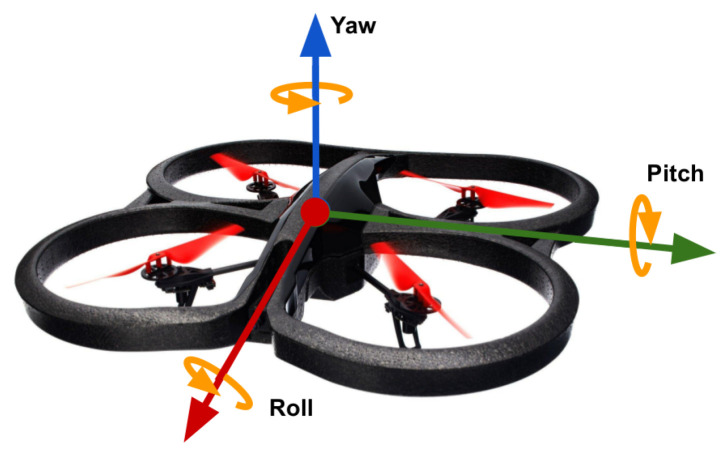
Quadcopter body frame: ϕ is the rotation on the X-axis, θ is the rotation on the Y-axis, and ψ is the rotation on the Z-axis.

To model the drone used in our simulations, we use a quadcopter model with 6 Degrees of Freedom (DoF), and is provided by the Gazebo simulator, available with the Robotic Operating System (ROS). This model is based on the AR.Drone model offered by the Parrot Company. Figure 2 shows a representation of the vehicle with the corresponding 3-D axes centered on the drone’s body frame. Rotation angles are indicated along each axis: roll (ϕ) tilts over the X-axis; pitch (θ) tilts over the Y-axis; and yaw (ψ) rotates over the Z-axis.

The model expects 4 flight commands (ϕ,θ,ψ,h). These commands represent: the angular position of the drone’s body frame in the pitch and roll angles whose values are given by (ϕ,θ), thus producing translation motion in those angles; rotational speed given by (ψ); and vertical speed given by (h). Note that the flight commands (ϕ,θ,ψ,h) are expected to have values in the range [−1,1].

The model also includes an inner loop controller that works to achieve the demanded values defined by (ϕ,θ,ψ,h). However, these values must be scaled first to obtain the angle positions and speed values in the corresponding units (degrees and m/s). This is done by multiplying the values given in (ϕ,θ,ψ,h) by predefined constant values: (1) the maximum tilt angle in roll; (2) maximum tilt angle in pitch; (3) the maximum rotational speed; (4) the maximum vertical speed, respectively. Note that this scaling process is carried out internally by the model’s software driver and the default maximum values are also set by the driver.

### 3.2. DeepPilot

In our previous work [7], we adapted the PoseNet architecture to predict only 2 drones flight commands (ϕ,θ). The achieved results were promising; nevertheless, the tests were conducted for zigzag tracks, where the gates had the same height and were placed in parallel to the drone’s camera plane. This meant that it was not necessary to predict the flight commands for (ψ,h). Instead, we use a PID controller to maintain height and a yaw angle. The latter posed the question of whether the 4 flight commands could be predicted by a CNN architecture.

Another motivation we had was that of investigating whether it would be possible to come up with a smaller network for the sake of saving processing time. Thus, inspired by the PoseNet architecture, in this work we propose a new network architecture whose main modules are based on Inception modules. We aim at extracting features from the input image to be finally evaluated with a regressor layer for flight command prediction. Furthermore, we realized that the regressor had a better performance if the output layers predicted unambiguous flight commands. For instance, we could train the modules to predict θ and ϕ flight commands, then create another model exclusively to predict ψ; and finally, another model to predict *h* only. Thanks to the flexibility of Keras and TensorFlow to load and run, in parallel, 3 models of the same architecture (the difference are the weights), we can predict the 4 flight commands (ϕ,θ,ψ and *h*); using a single image or a single mosaic image. We will refer to this architecture as DeepPilot now onwards.

**Figure 3:**
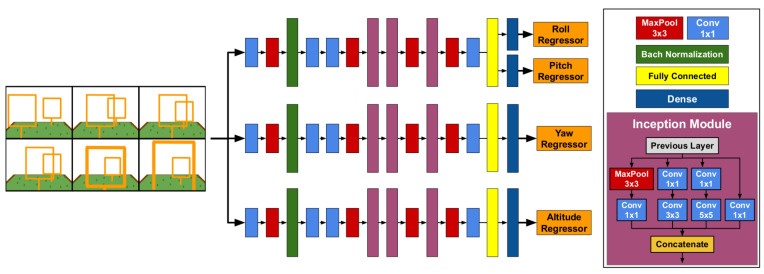
DeepPilot Architecture: our proposed DeepPilot runs 3 specialized models in parallel. The first one predicts ϕ and θ angular positions of the body frame; the second one predicts ψ, the rotational speed over the Z-axis; and the third one predicts *h*, the vertical speed. The size of the kernels is indicated in the colored boxes at the bottom-left.

Figure 3 depicts the new DeepPilot architecture. It is composed of 3 parallel branches aimed at generating the specialized models for the flight command prediction. Each branch has 4 convolutional layers with 3 inception modules, 1 fully connected layer and a regressor layer. The loss function used for each branch is shown in the Equation (1). Table 1 shows the parameters used to train DeepPilot.

(1) $$\begin{array}{c} loss\left(I\right)=\|\hat{x}-x{\|}_{2} \end{array}$$

where *x* corresponds to the flight command values for each image (I), recorded when piloting the drone manually, and $\hat{x}$ is the flight command predicted by the model. The loss function is evaluated 4 times, once for each control command: ϕ,θ,ψ, and *h*. Thus, our DeepPilot predicts values for (ϕ,θ,ψ,h) where each variable falls in the range of [−1,1].

### 3.3. Data Acquisition

**Figure 4:**
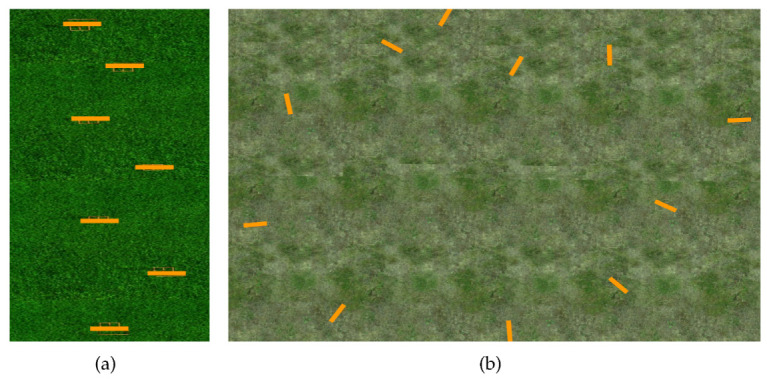
Racetracks in Gazebo for data collection. (**a**) This racetrack is composed of 7 gates 2 m in height. The track spans over a surface of 53.5 m × 9.6 m, and space in between gates from 10 m to 12 m. (**b**) A second racetrack composed of 3 gates 3.5 m in height, 4 gates 2 m in height and 4 gates 1.2 m in height, randomly positioned. The track spans over a surface of 72 m × 81 m, and space in between gates from 2 m to 12 m.

To create our image dataset, we manually flew the drone in two racetracks shown in Figure 4. For these racetracks, the positions, heights and orientations of the gates were chosen randomly. The goal was to provide different examples of what the gates would look like from the drone’s camera perspective. This included overlapping gates, gates with skew shape, gates at different heights, close and faraway gates, etc. Note that these tracks are different to those used later on in our experiments.

**Figure 5:**
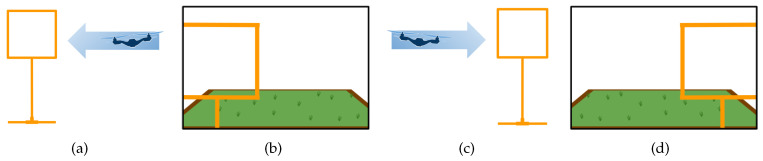
Schematic drone’s lateral motion: (**a**) outside view of the corresponding side motion when the gate appears to the left of the image (**b**); (**c**) corresponding side motion when the gate appears to the right of the image (**d**). The flight command ϕ will take values in the range of [−1,1]; values for (θ,ψ,h) will be set to zero.

**Figure 6:**
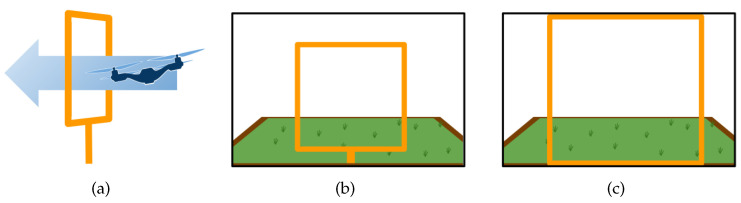
Schematic drone’s forward motion: (**a**) outside view of the corresponding forward motion when the gate appears in the image center (**b**,**c**). The flight command θ will take values in the range of [0,1]; values for (ϕ,ψ,h) will be set to zero.

**Figure 7:**
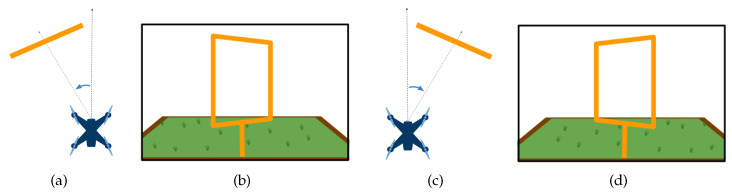
Schematic drone’s rotational motion: (**a**) outside view of the corresponding rotational motion in the yaw angle when the gate appears skewed towards the right of the image (**b**); (**c**) corresponding yaw motion when the gate appears skewed towards the left of the image (**d**). The flight command ψ will take values in the range of [−1,1]; values for (ϕ,θ,h) will be set to zero.

**Figure 8:**
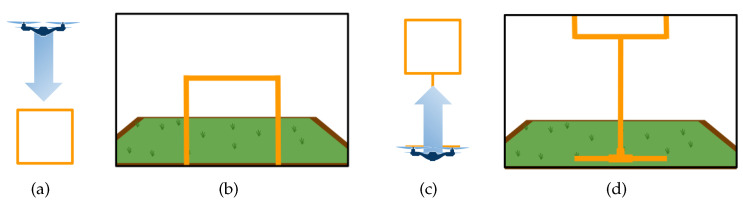
Schematic drone’s vertical motion: (**a**) outside view of the drone’s motion flying upwards when the gate appears at the bottom of the image (**b**); (**c**) downwards motion when the gate appears at the top of the image (**d**). The flight command *h* will take values in the range of [−1,1]; values for (ϕ,θ,ψ) will be set to zero.

We have acquired images to teach DeepPilot the 7 basic movements shown in Figure 5, Figure 6, Figure 7 and Figure 8, and also considering gates with variations in height and orientation. For each one of these images, a tuple of flight commands (ϕ,θ,ψ,h) is also recorded. These flight commands will be used as labels for the training stage, and as ground truth of the validation dataset.

Images were acquired aiming at representing seven basic motions: Lateral, forward, rotational, and vertical. Schematic representations of these motions are shown in Figure 5, Figure 6, Figure 7 and Figure 8. In the collected images, the gate was always kept in the camera’s viewing area. For each image, the flight commands given by the pilot are saved as the tuple (ϕ,θ,ψ,h). However, after the recording, a manual adjustment is made to the flight commands to identify the dominant drone’s motion as illustrated in the schematic figures. Once identified, the flight command corresponding to the dominant motion will be kept as recorded, whereas the other values in the tuple are set to zero. This was made to avoid ambiguous flight commands, which ultimately would be used as labels to our datasets.

**Figure 9:**
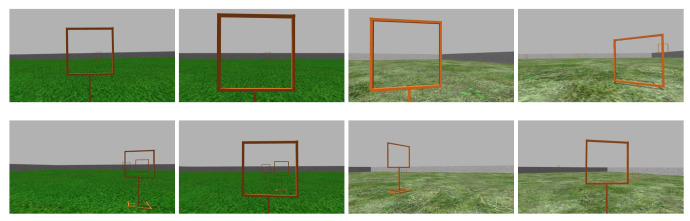
Examples of the dataset collected during the pilot’s flight.

As mentioned before, the data collection was carried out with the Gazebo simulator. We captured a total of 14,900 images with their respective flight commands (ϕ,θ,ψ,h) for each image, while the pilot controlled the drone manually. For training, we split the data up in 3 sub-datasets: the first one contains examples of lateral motions with flight commands for ϕ and θ; the second one includes forward motions with flight commands for ψ; and the third one contains vertical motions with flight commands for *h*. We used 13,050 images for training and 1850 for validation. Later these data were processed to generate the mosaic images, discussed in Section 3.4. Figure 9 shows some examples of dataset images. Table 2 summarizes the maximum and minimum values for (ϕ,θ,ψ,h). Each image has associated values in these ranges. In the training dataset, we use these values as labels, and as ground truth values for the validation dataset.

### 3.4. Mosaic Generation

The temporal analysis is one of the main contributions in this work to perform autonomous navigation through a racetrack. In several works, it is common to use a certain amount of frames to infer scenarios or actions in a scene [32], and for that purpose, a common practice is to use a stack of frames as input for a CNN architecture. However, that implied the addition of convolutional layers for each image to be stacked plus concatenate layers to extract the correlation between the stack frames. This implied a significant increase in the number of parameters that would grow as more images were used in the stack. Ultimately, this would increase the processing time as well. To get around this issue, we stitched frames to generate a mosaic image, which serves as input to CNN. We argue that a set of consecutive frames will encode a trend of the drone’s motion towards the gate, and such a trend can be captured in the position of the gate observed in each frame of the mosaic image. Furthermore, by using a mosaic image as input, it facilitates the use of a conventional CNN architecture that receives a single image with RGB channels.

**Figure 10:**
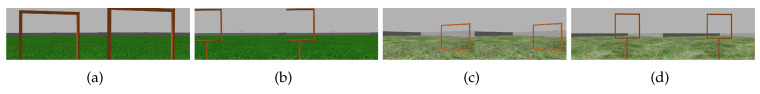
Mosaic image examples where each mosaic image (**a**)–(**d**) is composed of 2 frames.

**Figure 11:**
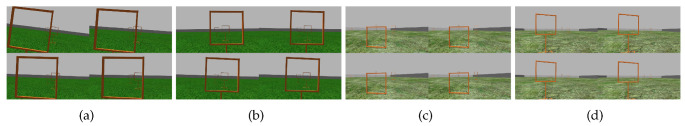
Mosaicimage examples where each mosaic image (**a**)–(**d**) is composed of 4 frames.

**Figure 12:**
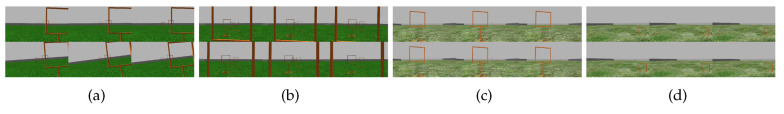
Mosaic image examples where each mosaic image (**a**)–(**d**) is composed of 6 frames.

**Figure 13:**
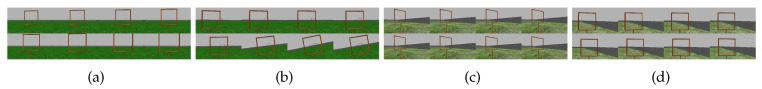
Mosaic image examples where each mosaic image (**a**)–(**d**) is composed of 8 frames.

To evaluate the efficiency of our strategy, we generated 4 new datasets from the sub-datasets described in Section 3.3. The first dataset contains mosaic images composed of 2 frames, see Figure 10; the second contains mosaic images composed of 4 frames, see Figure 11; the third contains mosaic images composed of 6 frames, see Figure 12; and the fourth contains mosaic images composed of 8 frames, see Figure 13. Every 5 frames an incoming frame is placed in one of the positions of the mosaic. When the mosaic is full, and for the next incoming frame, the image of the first position is discarded. The frames of the remaining positions are shifted to a previous location in the mosaic order, thus freeing space to allocate the new incoming frame. We chose to compose the mosaic with the sampling of every 5 frames, however, depending on the drone’s speed, this could be reduced to be carried out even in a frame-to-frame basis, or the opposite, every 10 or 15 frames if the drone’s motion is slower.

**Figure 14:**
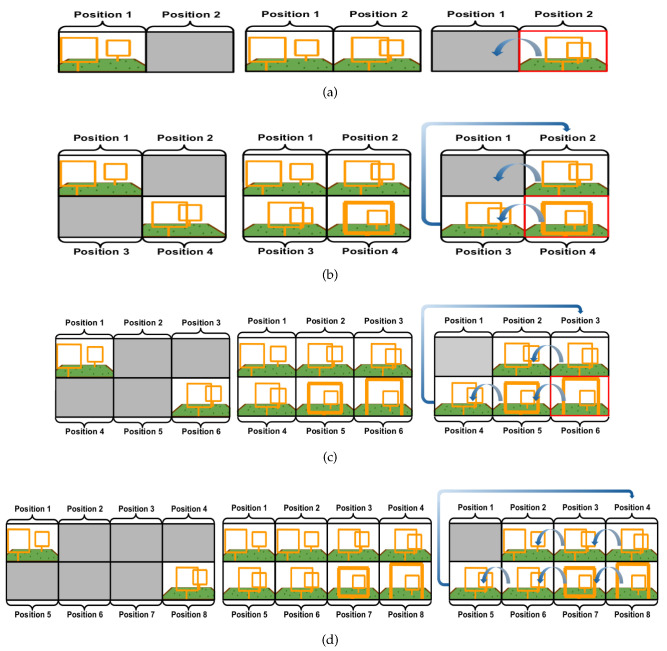
To compose the mosaic, the spaces in the mosaic image are filled every 5 frames: (**a**) Once the mosaic is full, the first frame in the mosaic is removed from position 1, and all frames are shifted to the left, this is, the frame in the position 2 moves to the position 1, and the frame in the position 3 moves to the position 2 and so on. (**b**) Illustrates the process for a mosaic composed of 4 images, note that the image in position 3 moves to position 2 when the shift occurs. (**c**,**d**) A similar process for when the mosaic is composed of 6 and 8 images, respectively.

Figure 14 shows the scheme described above to generate the mosaics for the case when the mosaic contains 6 frames. The images in the original datasets have a resolution of 640 × 360. Therefore the mosaics have the following dimensions: 1280 × 360, 1280 × 720, 1920 × 720 and 2560 × 720, for when the mosaic is composed of 2, 4, 6 or 8 frames respectively. However, the mosaics must be rescaled to a resolution of 224 × 224 to use them as input for DeepPilot network. From the main dataset composed of 14,900 images, a total of 41,360 mosaic images were generated. Table 3 summarizes the number of images used for training, and Table 4 shows the images used for validation, they indicate how many mosaics were generated for each control command.

### 3.5. Noise Filter

Before passing the flight commands predicted by the DeepPilot to the drone, 4 Exponential Average (EMA) filters were implemented. The EMA filters help to reduce the noise of the predicted flight commands, as well as to prevent oscillations and jolts. The equations are: Equations (2)–(5). Where alpha has a value of 0.1, (ϕ,θ,ψ,h) are the smoothed signals, respectively, $\left(\phi_{pred},\theta_{pred},\psi_{pred},h_{pred}\right)$ are the flight command prediction provided by DeepPilot. The value for alpha was chosen empirically, for the sake of space we present the value with the best smoothing result here.

(2) $$\begin{array}{c} \phi =\alpha *\phi +\left(1-\alpha \right)*\phi_{pred} \end{array}$$

(3) $$\begin{array}{c} \theta =\alpha *\theta +\left(1-\alpha \right)*\theta_{pred} \end{array}$$

(4) $$\begin{array}{c} \psi =\alpha *\psi +\left(1-\alpha \right)*\psi_{pred} \end{array}$$

(5) $$\begin{array}{c} h=\alpha *h+\left(1-\alpha \right)*h_{pred} \end{array}$$

## 4. Results and Discussion

In this section, we present our experimental framework to evaluate the performance of our proposed DeepPilot approach. First, we evaluate the Mean Squared Error (MSE) of DeepPilot to predict the 4 flight commands against ground truth using a validation dataset. Then, we compared the performance of DeepPilot against that of the PoseNet architecture; both trained to predict the 4 flight commands. We remind the reader that in our previous work [7], PoseNet was trained only to predict roll (ϕ) and pitch (θ). We used a zigzag racetrack simulated in Gazebo to carry out this evaluation. Finally, we present an evaluation, again in the Gazebo simulator, to assess the impact of using a mosaic image composed of 2, 4, 6 or 8 frames. Additionally, the best result of this evaluation was tested on other 2 racetracks to assess DeepPilot’s effectiveness to cross these racetracks autonomously. Note that all the racetracks used in these experiments were not used before to create the training datasets.

To run these experiments, we used an Alienware Laptop Computer with 16 GB in RAM and an Intel Core i7 running at 2.8 GHz, with an NVIDIA graphics card model GEFORCE GTX 1070. We used Ubuntu 16.04 LTS as the operating system and the Robotic Operating Systems (ROS) in its Kinetic Kame version. We used the Gazebo simulator version 7.0 in combination with the Tum_simulator package provided in [33] to enable the use of the AR.Drone vehicle, a drone of the Parrot company.

**Figure 15:**
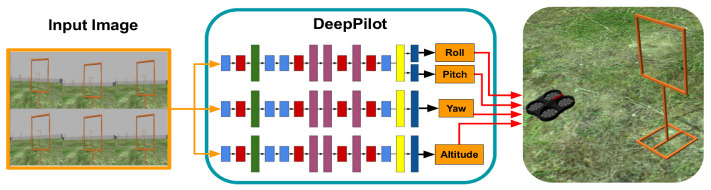
General control architecture in our approach based on an open-loop controller. The mosaic image corresponds to the observations of the world, in the racetrack. This image is passed to our CNN-based approach named DeepPilot to predict the flight commands (ϕ,θ,ψ,h), which are fed into the drone’s inner loop controller. As output, the drone will be commanded to fly towards the gate. This controller can be seen as a reactive controller.

We executed the experiments in the Gazebo simulations. Our Deep Pilot was run using a script in Python version 2.7. We used TensorFlow version 1.12.0 and Keras version 2.2.4. Camera frames were obtained at a frequency of 30 fps. For some experiments, every 5 frames are selected to compose a mosaic image. Once the mosaic image is complete, this is passed to DeepPilot whose output corresponds to a prediction of flight commands. These commands are smoothed out using the filter described in Section 3.5 and passed directly to the AR.Drone inner controller, see Figure 15. It is important to say that this inner controller is taken by us as a black box.

The drone’s inner controller requires only the 4 flight commands mentioned previously and its job is to maintain the flight at the demanded command. This means that our outer controller is an open-loop controller. It can also be considered as a reactive controller since no observation about the flight behavior of the drone is fed back into the controller. Note that there are not reference signals, nor we use any other onboard sensor such as the altimeter or the Inertial Measurement Unit (IMU).

### 4.1. DeepPilot Performance with a Validation Dataset

We have assessed the efficacy of DeepPilot using a validation dataset. This dataset was generated as explained in Section 3.3 and it contains 400 images. The objective in this evaluation is to assess the accuracy of the model learned by DeepPilot when trained to learn the flight commands ϕ,θ,ψ,h. As indicated before, the validation dataset is not part of the training dataset.

In our previous work [19], where PoseNet was trained for two flight commands only (ϕ&θ), we found that a mosaic of 6 frames produced more stable flight to cross the racetrack than when using a single image. For this reason, we have used a mosaic image of 6 frames as input.

Table 5 shows the MSE of the predictions returned by DeepPilot against the ground truth in the validation dataset. Note that the closest to zero the better; the maximum expected MSE would be 1. The results indicate that DeepPilot is likely to predict the 4 flight commands given a mosaic image where a gate is observed. This is a positive result, however, in our experience, the effectiveness of DeepPilot can be observed when tested in a racetrack. The next sections will present the obtained results in this regard.

### 4.2. DeepPilot vs PoseNet in a Zigzag Track

**Figure 16:**
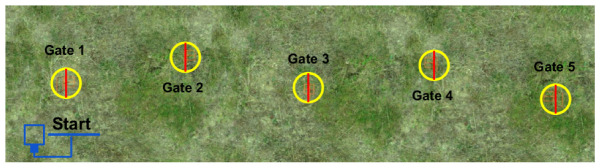
Zigzag racetrack in Gazebo to evaluate our proposed DeepPilot architecture and compared against PoseNet [6]. The track spans over a surface of 60m.×7m, with 11m. of space in between gates. We evaluated: (1) PoseNet trained to predict the 4 flight commands (ϕ,θ,ψ,h); (2) PoseNet trained to predict only (ϕ,θ); (3) DeepPilot trained to predict the 4 flight commands. We used a mosaic image composed of 6 frames as image input to the networks.

We have compared the performance of PoseNet [7] against our proposed DeepPilot performing in zigzag racetrack shown in Figure 16. The track was composed of 5 gates with the same height and with parallel position. In [7], we experimentally showed that using a single image as input to the network produced oscillations in the prediction and hence, unstable flight. For this reason, in these experiments we use a mosaic composed of 6 frames as input to the networks.

**Figure 17:**
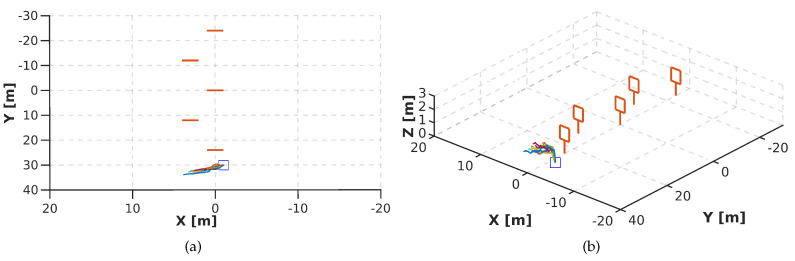
Top view (**a**) and side view (**b**) of the performance of PoseNet, trained to predict the 4 flight commands, for 10 runs in the zigzag racetrack. Note that PoseNet failed to cross the first gate in all runs. We used a mosaic image composed of 6 frames as image input to the network.

For our first experiment, we trained PoseNet to learn the 4 flight commands (ϕ,θ,ψ,h). However, when the 4 flight commandss are used in the regression, the network did not work correctly. Figure 17 shows that the drone fails to complete the track. We attribute this to the lack of examples to differentiate ambiguous flight commands. For instance, for the same image, lateral motion or rotation in ψ could have the same possibility to be predicted. This causes a constant oscillation that stops the drone from flying toward the gate, see Figure 17.

**Figure 18:**
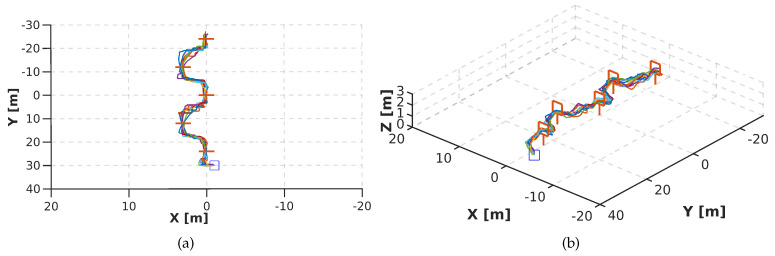
Top view (**a**) and side view (**b**) of the performance of our proposed DeepPilot, trained to predict the 4 flight commands (ϕ,θ,ψ,h), for 10 runs in the zigzag track, each run was successful. The average time for the drone to complete the track was 2 min 31 s and the command prediction output ran at 25 fps.

Figure 18 shows the performance of DeepPilot to predict the 4 flight commands. Note that the autonomous flight has significantly been improved. We performed 10 runs, where the average time to the drone completed the track was 2 min 31 s, and the command prediction frequency for ϕ,θ,ψ,h was 25 fps. Figure 18b shows the side view of the runs. It can be seen that DeepPilot keeps a constant *h* and no peaks are found when crossing the gate. Also, Figure 18a showed some runs when the drone was not kept perpendicular to the gate to perform lateral movements. In this case, the drone was oriented towards the gate and moved forward. Finally, Table 6 shows the processing time for these experiments, for both PoseNet and DeepPilot. The latter run at a faster speed with 25.4 fps than PoseNet with 21.3. These results are encouraging since we sought to reduce the processing time by proposing a more compact architecture with less neural layers.

### 4.3. Mosaic Evaluation

**Figure 19:**
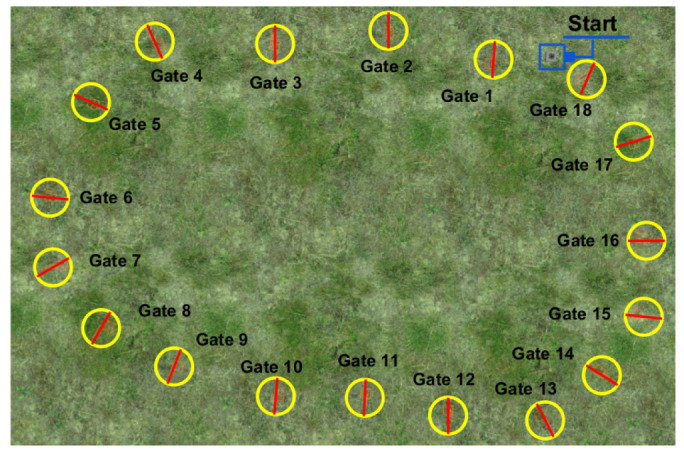
First racetrack to evaluate the mosaic with a total of 18 gates. The track spans over a surface of 62 m × 44 m, and the space in between gates from 7 m to 9 m. The gates’ height are as follows: 7 gates 2 m in height; 8 gates 2.5 m in height; and 3 gates 3 m in height, randomly positioned. Note that this track was not used for training. A video illustrating the performance of our proposed DeepPilot in this racetrack can be found at https://youtu.be/Qo48pRCxM40.

In this section, we present the evaluation of the DeepPilot network using mosaics, composed of 2, 4, 6 and 8, as input. For this purpose, we designed a complex track, with narrow gates, compared to other race tracks, which is formed by 7 gates of 2 m height, 8 gates of 2.5 m height and 3 gates of 3 m height randomly positioned, see Figure 19. The gates’ frame measures 1 × 1 m, and the height varies from 1 m, 1.5 m and 2 m. This evaluation aims to find the best option to provide the DeepPilot network with information about the previous states.

All the networks using the different mosaic variations were trained with the same training parameters: (1) Adam Optimizer; (2) 500 epoch; (3) batch size 32; (4) ReLu as an activation function. They also generated 3 sub-datasets for each mosaic (2, 4, 6 and 8) from the main dataset, i.e., 12 sub-datasets.

**Figure 20:**
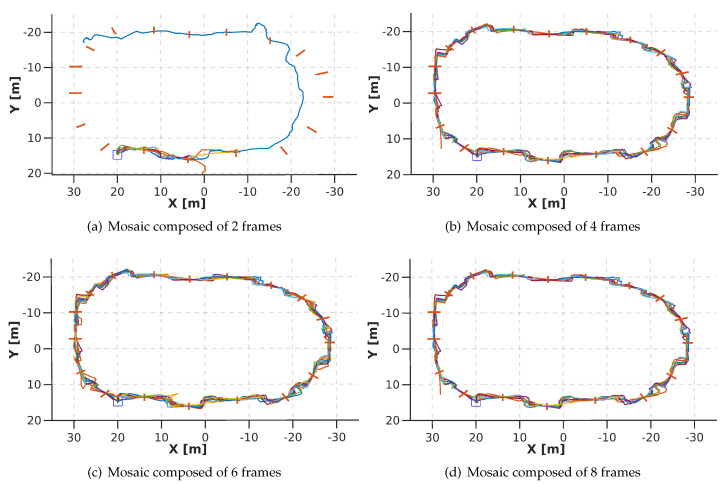
Top view of 10 runs performed on the track shown in Figure 19. Each sub-image shows the DeepPilot’s performance when using a mosaic image composed of: (**a**) 2 frames; (**b**) 4 frames; (**c**) 6 frames; (**d**) 8 frames. Note that (**c**,**d**) are successful and similar in performance.

**Figure 21:**
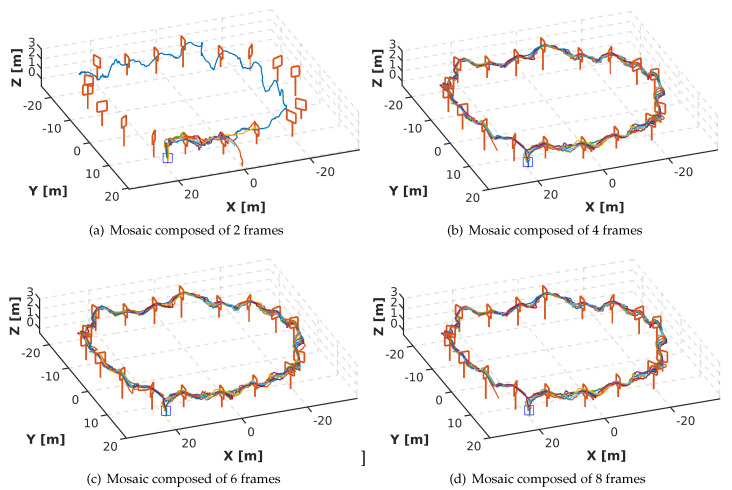
Side view of the 10 runs shown in Figure 20. Note that using mosaic images of 2 or 4 frames are unstable and do not allow for the drone to finish the racetrack.

In Figure 20, the trajectories are shown from a top view. Figure 20a corresponds to the training using a mosaic image, composed of 2 frames, as input. The performance of this network is stable in *h*, see Figure 21a. However, it was not able to cross more than 3 gates. Even on the longest run (purple line in Figure 20a the drone does not cross from gate 4 to gate 8 (rotation section), then the drone joins the track at gate 9 (Zigzag section), but from gate 13 the drone fails again.

The ψ flight command is the most complex of the four commands, as it depends on the above information to determine whether to keep turning or stop. In the case of gates with oblique positions, it is easy to lose it in the viewing area and without a global localization the drone will not be able to align itself perpendicularly with the gate. When the ψ prediction fails, we attribute it to the lack of enough input information for the network to predict the required ψ angle in time.

Figure 20b corresponds to the training using a mosaic image, composed of 4 frames, as input. The performance of this network is not stable in any of the flight commands. The values provided by the network cause the drone to oscillate at the same point, preventing the drone from crossing the gate. This is also detrimental to *h*, since the images produced by the oscillation indicate that it should go up. This is why Figure 21b shows peaks in the *h*.

Figure 20c,d correspond to the training using 6 and 8 images to compose the mosaic image, respectively. The performance of both networks is similar and quite stable in the 4 flight commands. Figure 21c,d is shown a side view of the trajectory. These show the height changes that the drone performs according to the gate. The mean time per lap is 7.6 min for the mosaic composed of 6 frames and 7.9 min for the mosaic composed of 8 frames.

Based on the results obtained in the previous evaluation, we decided to use the mosaic image, composed of 6 frames, as input for the DeepPilot network, since it generated the most stable result. By using 6 frames, we avoid losing resolution, and it does not require much time to generate the mosaics in comparison with the mosaic composed of 8 frames.

**Figure 22:**
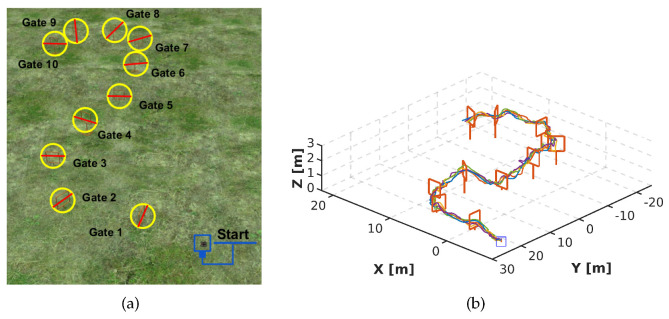
Second racetrack to evaluate DeepPilot using a mosaic of 6 frames. The track spans over a surface of 50 m × 23 m, and the space in between gates goes from 5 m to 9 m. Note that this track was not used for training. (**a**) The track is formed by 10 gates: 4 gates of 1.2 m height and 6 gates of 2 m height. (**b**) Drone’s trajectories obtained for 5 runs for this racetrack. DeepPilot piloted the drone in the track providing the flight commands (ϕ,θ,ψ,h). A video illustrating the performance of our proposed DeepPilot in this racetrack can be found at https://youtu.be/Qo48pRCxM40.

**Figure 23:**
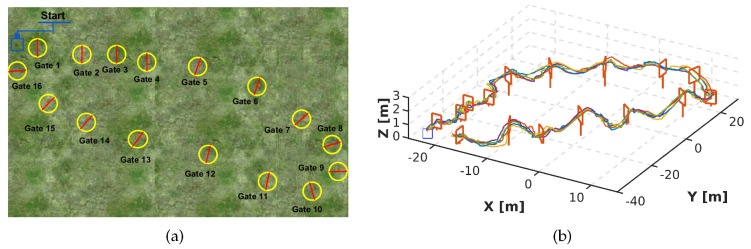
Third racetrack to evaluate DeepPilot using a mosaic of 6 frames. The track spans over a surface of 70 m × 40 m, and the space in between gates goes from 6 m to 15 m. Note that this track was not used for training. (**a**) The track is formed by 16 gates: 8 gates of 1.2 m high and 8 gates of 2 m high. (**b**) Drone’s trajectories obtained for 5 runs for this racetrack. DeepPilot piloted the drone in the track providing the flight commands (ϕ,θ,ψ,h). A video illustrating the performance of our proposed DeepPilot in this racetrack can be found at https://youtu.be/Qo48pRCxM40.

To show the efficacy of DeepPilot, we created a second and third track in Gazebo, and similar to the first racetrack, these other 2 tracks were also previously unseen to DeepPilot. We varied position, height and orientation of the gate. The second track is composed of 10 gates; 4 gates of 1.2 m height and 6 gates of 2 m height, see Figure 22a. In this track, 5 runs were made, which were successfully completed. In Figure 22b, show slight oscillations; this is due to the gates being close to each other. The mean time per run is 4.7 min. The third track is composed of 16 gates; 8 gates of 1.2 m height and 8 gates of 2 m height, see Figure 23a. In this track, also 5 runs were carried out with the drone performing orientation changes without collision, and constant height changes with few oscillations, see Figure 23b. The mean time of the 5 runs was 9.2 min.

### 4.4. Discussion on Porting Over to the Physical Drone

Our proposed DeepPilot architecture was tailored to predict 4 flight commands identified as ϕ,θ,ψ,h. This choice was not arbitrary. It is based on the standard input for inner controllers such as those used by the AR.Drone or the Bebop vehicles from the Parrot Company.

As we have described before, the Gazebo simulator provides the AR.Drone model, which receives the 4 flight commands used in this work and enables us to observe the flight dynamics for a given tuple of flight commands.

According to the results we have presented in this work, DeepPilot can effectively fly the drone to cross the racetrack successfully. Therefore, our next step is to test this concept with the physical vehicle, and for that, our first choice is to use the Bebop 2.0 Power Edition (PE). A drone that uses the same flight commands, with a speed of 16m/s and with a Software Development Kid that enables software implementation on ROS.

From the above, a first step could be that of using the DeepPilot’s model (learned in simulation) directly with the Bebop vehicle. For that, we only need to change the topic that subscribes to the Bebop’s image publisher and the topic that publishes the flight commands to the Bebop’s command subscriber.

However, it is expected that the environment of the racetrack changes significantly from the one used in the simulations. Primarily because Gazebo does not provide photo-realistic scenarios. In order to address this issue, we are planning to carry out two steps: (1) collect a small image dataset of our physical gates in the real test environment. The gates could be placed randomly, with different heights and different orientations; (2) We could train from scratch with this new dataset. Instead, we would use the weights learned in simulation as initial weights for the new training. In our experience, this would accelerate convergence.

A final issue is related to the use of GPU. Currently, the Bebop PE does not have enough computational power to run TensorFlow or Keras. However, in our previous work [1], we have managed to mount an external computer to process an outer controller or any other software program. Thus, we could mount an embedded computer with GPU such as the Jetson TX2 from NVIDIA. We confirm that all the operating system and software tool used in this work can be loaded into this board. In the extreme, the Bebop PE supports image and control command transmission via WiFi using ROS. This allows us to use a Laptop with GPU as a Ground Control Station, where we could run all our software with the caveat that this solution depends on the WiFi range.

Therefore, we argue that porting over to the physical platform, either the Bebop or any other platform with similar flight commands, is a seamless and straightforward process. This will be part of our future work.

## 5. Conclusions

We have presented DeepPilot, a CNN architecture capable of piloting a drone to navigate autonomously through a racetrack. DeepPilot receives only camera images as input and predicts flight commands. These commands represent: the angular position of the body frame in the roll and pitch angle; the rotational speed in the yaw angle; and the vertical speed. This is equivalent to the 4 flight commands generated by a human pilot using a joystick controller.

In addition, our proposed approach has been evaluated in a simulation environment with different racetracks. Gates at different heights and orientations form challenging tracks to be traversed by the drone autonomously. We have assessed the performance of DeepPilot using a validation dataset, but also by carrying out several flights in the racetracks, previously unknown to the network, in order to appreciate the repeatability of DeepPilot.

We have also shown the impact of the temporality factor; this is, the use of consecutive frames stitched together to form a mosaic image that is passed as input to the network. The advantage of this approach versus a stack of frames is that a mosaic image enables us to keep using a single image with RGB channels as input without major handcrafting of the network. We have evaluated mosaics composed of 2, 4, 6 and 8 frames, and the results show that the mosaic of 6 frames produces the best results. In average DeepPilot process at 25 frames per second.

The proposed methodology behind DeepPilot paves the way towards the development of an artificial pilot seeking to exploit similar capabilities to those used by human pilots in drone racing, namely: experience; memory; and reactive behavior. For the latter, our approach can be seen as an imitation approach where DeepPilot learns from the images and flight commands recorded during a manual flight. In this sense, we have shown that DeepPilot is capable of predicting the flight commands given the input image.

From the above, we are interested in testing DeepPilot in real scenarios. Given that our approach is purely based on DL, porting over to the drone’s onboard computer can be done seamlessly, independently of whether GPU is available or not. Hence, we are in the process of building a large outdoors racetrack, similar to what has been presented in our simulations. Furthermore, we will continue investigating possible ways of improving the architecture, also seeking to increase its processing speed. This could enable the drone to fly at a faster speed, but also to process image data acquired with high-speed cameras, which could prove useful for agile and aggressive flights, expected in drone racing.

Finally, we are prepared to make public the datasets we have generated for training and validation, our trained DeepPilot model, the racetracks in Gazebo, and the python scripts to execute our model.

## Figures and Tables

**Table 1:** Parameters used to train 3 specialized models in our DeepPilot approach to learn the flight commands represented by the variables roll and pitch (ϕ,θ), yaw (ψ), and altitude (*h*).

DeepPilot Models	Parameters	Value
(1) Roll & pitch (2) Yaw (3) Altitude	Optimizer	Adam
	Epoch	500
	Batch size	32
	Activation function	ReLu
	Learning rate	0.001

**Table 2:** Distribution of ground truth flight command values associated as labels to the images in the training and validation datasets recorded during a manual flight.

Command	Training Flight Command				Validation Flight Command			
	Mean	Std	Max	Min	Mean	Std	Max	Min
Roll	0.028	0.296	± 0.9	± 0.1	0.053	0.299	± 1	± 0.1
Pitch	0.232	0.357	+ 1	+ 0.1	0.696	0.422	+ 1	+ 0.1
Yaw	0.0	0.036	± 0.1	± 0.05	0.005	0.037	± 0.1	± 0.05
Altitude	0.0023	0.1409	± 0.1	± 0.05	0.0013	0.0925	± 0.1	± 0.05

**Table 3:** Number of mosaic images in the datasets used to train the models for the flight commands (ϕ,θ,ψ,h).

Datasets	ϕ & θ	ψ	*h*	Resolution
mosaic composed of 2 frames	5298	2928	938	1280 × 360
mosaic composed of 4 frames	5296	2926	936	1280 × 720
mosaic composed of 6 frames	5294	2924	934	1920 × 720
mosaic composed of 8 frames	5292	2922	932	2560 × 720

**Table 4:** Number of mosaic images in the datasets used to evaluate the models for the flight commands (ϕ,θ,ψ,h).

Datasets	ϕ & θ	ψ	*h*	Resolution
mosaic composed of 2 frames	508	448	238	1280 × 360
mosaic composed of 4 frames	506	446	236	1280 × 720
mosaic composed of 6 frames	504	444	234	1920 × 720
mosaic composed of 8 frames	502	442	232	2560 × 720

**Table 5:** MSE and its standard deviation (Std) for the prediction of the flight commands ϕ,θ,ψ,h using DeepPilot. A mosaic image composed of 6 frames was used as input. The network was trained with 5000 images and evaluated with 400 images.

Image Input	MSE θ	Std	MSE ϕ	Std	MSE ψ	Std	MSE *h*	Std
Mosaic_6	0.139	0.372	0.144	0.370	0.0135	0.0965	0.0029	0.0507

**Table 6:** Mean processing time expressed in frames per second (fps) and its standard deviation (Std) for PoseNet [6] and DeepPilot in the zigzag racetrack. A mosaic image composed of 6 frames was used as input.

	Mean fps	Std
PoseNet - Mosaic_6	21.3	0.1761
DeepPilot - Mosaic_6	25.4	0.1100
